# Retraction: Isolation and molecular characterization of extended spectrum beta lactamase producing Escherichia coli from chicken meat in Pakistan

**DOI:** 10.1371/journal.pone.0349235

**Published:** 2026-05-13

**Authors:** The PLOS One Editors

After publication of this article [1], several concerns were raised about the results presented in Figs 1, 3, and 6, and Table 5. Specifically,

- The flowcharts in Fig 1 of [1] and in Fig 1 of [2] are the same.
- The PCR gels in Figs 3 and 6a-i of [1] and Figs 4 and 5a-i of [2] appear similar to each other, respectively.
- The ladder and the remaining PCR bands in all panels from Fig 6a to 6i appear similar to each other.
- When levels are adjusted to visualize the background of the PCR gels in Fig 3 and Fig 6, there are regions that appear discontinuous with the adjacent background in the ladder area of the gels.
- Table 5 reports MIC50 = 0.19 μg/mL, below the stated range of 0.25–1 μg/mL

During editorial follow-up, the corresponding author stated that both articles [1] and [2] originated from the same overarching project, sharing a similar methodological framework, and confirmed that for this reason the methodological flow charts in Fig 1 of each article are the same; however, they stated that the PCR gels presented in the two articles originate from bacterial isolates collected in different districts of Khyber Pakhtunkhwa, Pakistan. The corresponding author also indicated that the similarities between different PCR gel images arose from the use of the same PCR-based protocol and identical imaging conditions for the identification of the same bacterial strain (*E. coli*) in different isolates.

Regarding the background anomalies in the PCR images, the corresponding author stated that the issues above resulted from artefacts introduced during image processing. Some underlying data for the panels of concerns were provided during editorial follow-up with the authors; however, these data were insufficient to resolve the concerns listed above.

Regarding Table 5, the corresponding author stated that MIC₅₀ was correctly reported as 0.19 µg/mL because it was calculated as a numerical median, whereas the MIC range represents the observed MIC values among individual isolates. However, the *PLOS One* Editors determined that this explanation did not resolve the issue.

In light of the above issues, which raise concerns about the reliability and integrity of the reported results, the *PLOS One* Editors retract this article.

IK, YA, and EHA did not agree with the retraction. ZL and SA responded but expressed neither agreement nor disagreement with the editorial decision. LM either did not respond directly or could not be reached.

Figs 1, 3, and 6 of [1] appear similar to Figs 1, 4, and 5 previously published in [2] under a CC BY-NC-ND license by Elsevier in 2022 and remain subject to the license that applied to the original article.
